# Association between Urinary N-Acetyl-Beta-D-Glucosaminidase and Microalbuminuria in Diabetic Black Africans

**DOI:** 10.1155/2012/235234

**Published:** 2012-08-26

**Authors:** Francis Patrick Udomah, Udeme Ekpenyong Ekrikpo, Emmanuel Effa, Babatunde Salako, Ayodeji Arije, Solomon Kadiri

**Affiliations:** ^1^Department of Medical Laboratory Sciences, University of Calabar Teaching Hospital, Calabar, Nigeria; ^2^Nephrology Unit, Department of Internal Medicine, University of Uyo Teaching Hospital, Uyo, Nigeria; ^3^Nephrology Unit, University of Calabar Teaching Hospital, Calabar, Nigeria; ^4^Department of Internal Medicine, University College Hospital Ibadan, Ibadan, Nigeria

## Abstract

Diabetes mellitus is the commonest cause of ESRD worldwide and third most common cause in Nigeria. Recent reports from Nigeria indicate the prevalence of diabetic nephropathy as an aetiology of ESRD is increasing necessitating early diagnosis of diabetic nephropathy. We measured the urinary excretion of N-acetyl-beta-D-glucosaminidase (NAG), NAG/creatinine ratio, urinary protein-creatinine ratio and calculated eGFR in 30 recently diagnosed nonhypertensive diabetics and 67 controls. The age and sex distribution, systolic blood pressure, serum and urinary creatinine were similar for both groups. There was higher urinary excretion of NAG (304 versus 184 *μ* mol/h/L, *P* < 0.001) and NAG/creatinine ratio (21.2 versus 15.7 *μ* mol/h/L/mmolCr, *P* < 0.001) in the diabetics than controls. There was a strong correlation between NAG/creatinine ratio and albumin/creatinine ratio (*r* = 0.74, *P* < 0.001). A multivariate linear regression model showed a significant linear relationship between NAG/creatinine ratio and albumin/creatinine ratio after adjusting for the effect of blood pressure, age, sex, and serum creatinine. The strong association found between albumin/creatinine ratio and NAG/creatinine ratio perhaps indicates the need for further investigation of the clinical utility of NAG/creatinine ratio as a screening tool for early nephropathy in African diabetics.

## 1. Introduction

End-stage renal disease is on the increase worldwide. However, it is difficult to appropriately compare international data on the aetiology, incidence, and prevalence because of differences in how data for various registries are derived, different patient demographics, and quality of healthcare among others. Diabetes mellitus (DM) is still recognized in the US and Europe as the commonest cause of end-stage renal disease (ESRD). Indeed recent data from the US Renal Data System suggests that the rates of ESRD due to DM and hypertension rose by 2.2% and 2.7%, respectively, in 2009 with overall prevalent ESRD estimated at 1,738 per million population [1]. African Americans were in the majority. Reports from Europe and Asia have also shown a rise in the incidence of ESRD over the years [2–4]. In Nigeria, many hospital-based reports put diabetic nephropathy as the third most common cause of ESRD [5–7], but it appears that the proportion of ESRD caused by diabetic nephropathy is increasing [8].

In the last two decades, studies have focused on the role of glomerular injury in early diabetic nephropathy (as measured by the onset of persistent microalbuminuria), but attention is now being shifted to a concurrent or perhaps earlier occurrence of tubular injury in diabetic nephropathy [9]. For instance, tubular hypertrophy and reduced organic ion transport in the proximal tubules are apparent even before the onset of overt proteinuria in diabetics [10]. Several urinary markers with clinical utility in the prediction of early nephropathy including transferrin, type IV collagen, alanine aminopeptidase, and *N*-acetyl- *β* -D-glucosaminidase (NAG) have been identified [11].

NAG is a high-molecular-weight (140,000–160,000 Da) lysosomal enzyme that cannot pass into the glomerular ultrafiltrate because of its size. It has been extensively studied as a marker of renal tubular injury and is known to leak into the tubular fluid from the proximal tubular cells when injured [12]. However, human and animal studies have suggested that urinary NAG levels may indicate a functional tubular disorder rather than tubular damage [13, 14]. Studies have demonstrated a significant increase in urinary NAG excretion in type 2 diabetics compared with controls [15–17]. A progressive rise in the levels corresponding to poor glycemic control has been noted [15] with reduced levels seen after good glycemic control [18]. In the UKPDS study, NAG levels at diagnosis were much higher in diabetics compared to albumin excretion and it appears that the levels are elevated even in those with normoalbuminuria. [19]. This may suggest that tubular damage in diabetic nephropathy appears to be independent of glomerular injury. Early renal dysfunction may be predicted by the early rise in NAG in diabetes as the majority of patients may also show glomerular hyperfiltration [20] and increased urinary albumin excretion [21].

The objective of this study was to determine the clinical utility of urinary NAG excretion with particular reference to the degree of association between it and microalbuminuria in African diabetics.

## 2. Methodology

Thirty (30) nonhypertensive diabetics (less than 3 years since diagnosis) who did not have features of urinary tract infection were recruited into the study. The controls consisted of 67 volunteers from the community in which the hospital is situated with no history or clinical features suggestive of hypertension, diabetes mellitus, nor urinary tract infection. These were matched by age to the subjects. None of the participants were on angiotensin-converting enzyme inhibitors or angiotensin receptor blockers. All pregnant women were also excluded from the study.

About 10 ml of random urine samples were obtained from all the subjects and controls, and urinary NAG activity was determined using the colorimetric method of Yuen et al. [22] which was the adapted procedure for the urinary NAG kit obtained from PPR Diagnostics Ltd, London. The tests were not run in duplicates because of the increased cost this would confer on the study in a resource-poor setting. Urinary and serum creatinine was measured by the Jaffe kinetic method. Total urinary protein was determined by the trichloroacetic acid (TCA) turbidimetric method. The average of two blood pressures taken to the nearest 2 mmHg was recorded for each individual. Standardization of NAG excretion was done by computing the urinary NAG/creatinine ratios for all the study participants. The estimated GFR for each participant was computed using the 4-variable MDRD equation [23].

A comparison of sociodemographic and clinical characteristics was undertaken using the student *t*-test (or its nonparametric equivalent) for continuous data and Pearson's chi square for categorical data. Univariate linear regression models were used to identify the degree of association between urinary albumin/creatinine ratio and NAG/creatinine ratio. Factors with a *P* value of less than 0.25 at the univariate model were included in the multivariate model using a forward selection process. The effect of age and blood pressure on the relationship between urine albumin/creatinine ratio and NAG/creatinine ratio was considered clinically important enough to warrant inclusion of these factors in the multivariate model. Model diagnostics were then performed. All analyses were performed using STATA 10 (StataCorp, Texas, USA).

Ethical approval was obtained from the University of Calabar Human Research Ethics Committee.

## 3. Result

There were 30 nonhypertensive diabetics and 67 controls who participated in the study. The mean age of the study participants was 37.4  ±  9.5 years.  Table 1 summarizes the sociodemographic and clinical characteristics of the diabetics and controls.

The median estimated GFR for the diabetics (85.1 mL/min, IQR 45.4–111.8 mL/min) and controls (94.3 mL/min, IQR 73.8–127.7 mL/min) was significantly different while the median urinary albumin/creatinine ratio was 6.18 mg/mmol creatinine (IQR 4.68–9.42 mg/mmol creatinine) for the diabetics and 2.09 (IQR 1.51–2.81 mg/mmol) for the controls. The diabetics had a lower eGFR and higher urinary albumin/creatinine ratio than the controls (Table 2).

## 4. Tubular Function in Both Groups

**Figure 1:**
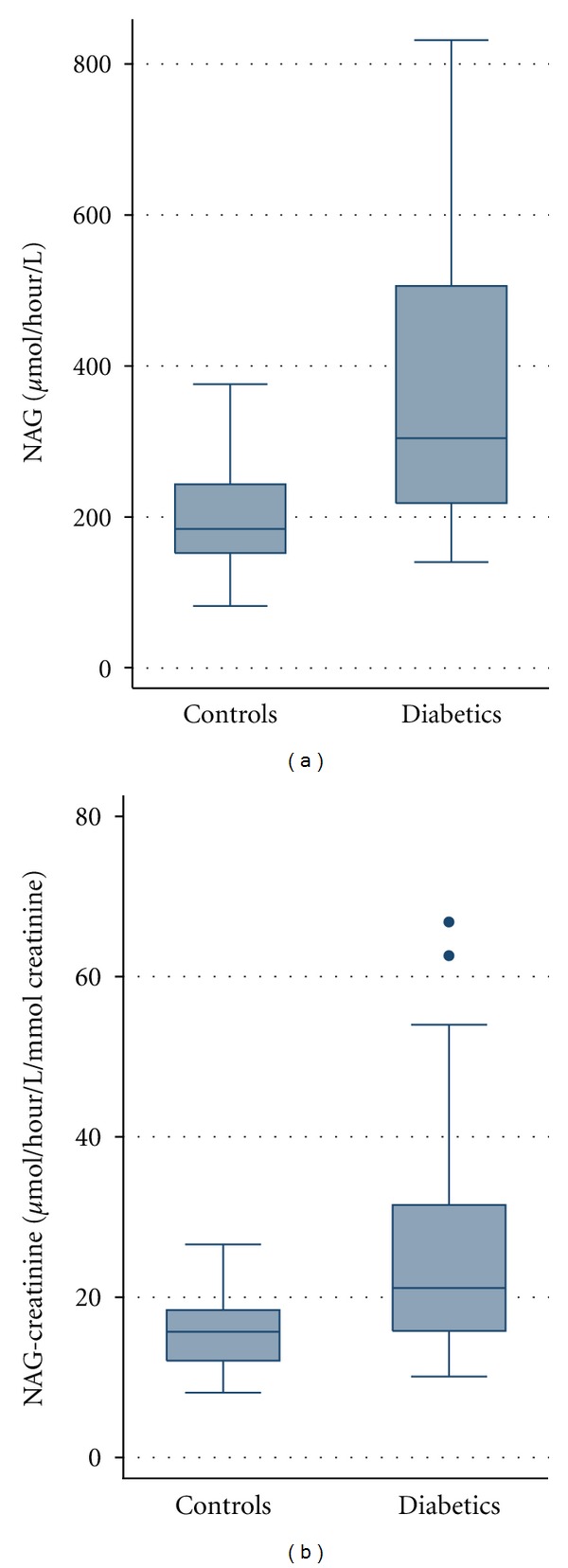
Comparison of NAG/NAG-creatinine ratios in both study groups.

The urinary NAG excretion for both groups was standardized by finding the urinary NAG-creatinine ratios for each participant. The median urinary NAG (304 versus 184 *μ*mol/h/L, *P* < 0.001) and NAG-creatinine activity (21.2 versus 15.7 *μ*mol/h/L/mmolCr, *P* < 0.001) in diabetics was significantly higher than that in the controls. This is shown in Figure 1.

## 5. Relationship between NAG/Creatinine Ratio and Urine Albumin/Creatinine Ratio

**Figure 2:**
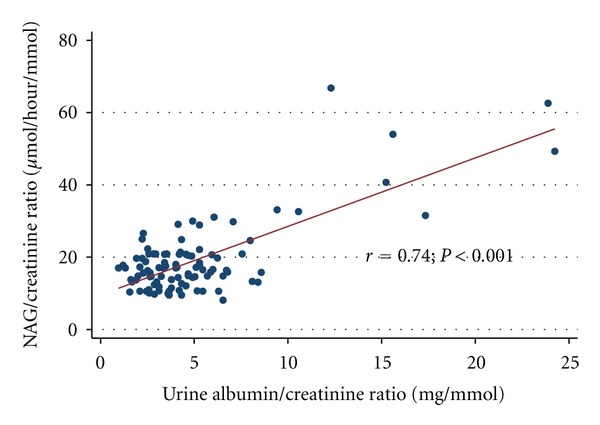
Linear relationship of urinary NAG/creatinine ratio to urinary albumin/creatinine ratio.

There was a significant positive correlation between the NAG/creatinine ratio and urinary albumin/creatinine ratio (*r* = 0.74, *P* < 0.001), Figure 2. There was also significant negative correlation between urinary NAG/creatinine ratio and systolic blood pressure (*r* = −0.21, *P* = 0.04) but no correlation between NAG/creatinine ratio and eGFR (*r* = 0.44, *P* = 0.06).

A multivariate linear regression model was employed to assess the relationship between urinary albumin/creatinine ratio and NAG/creatinine ratio after adjusting for the effect of other factors which may influence changes in urinary albumin excretion. Table 2 shows that there exists a positive linear relationship between urinary albumin/creatinine ratio and NAG-creatinine ratio after adjusting for gender differences, changes in systolic and diastolic blood pressures, serum creatinine, age, and diabetes mellitus status.

## 6. Discussion 

Diabetes mellitus is a significant global public health problem. In the low-income countries of sub-Saharan Africa, it is important to detect and institute measures to prevent the onset and progression of overt nephropathy in diabetics because the cost of renal replacement therapy is beyond the reach of most individuals in this region. Added to the economic, social, and psychological burden of care is the high morbidity and mortality accompanying it, especially in those with long term poor glycemic control. The ability to detect early and likely reversible renal injury using relatively inexpensive, noninvasive, and reliable biomarkers should lead to better care. Diabetic tubulointerstitial injury is a feature of early diabetic nephropathy and an important predictor of future renal dysfunction [24]. Before the onset of gross structural changes in the renal tubules, lysosomal enzymes like NAG have been found to be markedly increased in urine [24]. Several studies elsewhere have shown increased urinary excretion of NAG in diabetics [25–27]. This has been corroborated by our study. 

A positive linear relationship between NAG/creatinine ratio and ACR demonstrated in this study has been noted in other studies [15, 18, 27] where it appears it is statistically independent of glycemic control [27]. This may suggest some utility of NAG/creatinine ratio in the early diagnosis of diabetic nephropathy. An analysis of urine samples obtained from the Diabetes Control and Complications Trial (DCCT) has shown that the occurrence of elevated urinary NAG excretion independently doubled the risk of macroalbuminuria in later life. There was also nearly a twofold increased risk of developing microalbuminuria in later life [21]. The use of angiotensin-converting enzyme inhibitors has also been found to reduce urinary NAG and albumin excretion [26, 28] suggesting a close relationship between urinary NAG and albumin levels. Indeed, even after adjusting for the effect of blood pressure, age, sex, and serum creatinine levels, NAG/creatinine ratio still had a strong positive linear relationship with ACR in this study.

We also found significant correlation between urinary NAG excretion and serum creatinine levels. This is corroborated by other studies elsewhere [28] and may mean that, in the absence of glomerular disease, tubular dysfunction leading to increased urinary NAG excretion may also be associated with impaired tubular handling of creatinine. The lack of correlation between NAG/creatinine ratio and eGFR may be due to the relatively small sample size.

Contrary to observations in other studies [29], age and gender did not seem to influence the levels of urinary NAG. This may be because of the small number of subjects in the study. It would appear that sugar control for the majority of our diabetics was suboptimal hence the increased levels of urinary NAG. NAG levels are thought to reflect glycemic control especially in type 1 diabetics [25]. Whether increased levels are transient and related to equally transient hyperglycemia is unclear as we did not measure HBA1c levels since this was not a routine test in our center at the time of this study.

## 7. Conclusion

This study, probably the first in Black Africa, showed that urinary NAG levels are elevated in African diabetics and correlate strongly with ACR. Demonstrating that this occurs earlier than microalbuminuria may suggest its usefulness as a marker for screening our diabetic patients for early renal disease. There is therefore a need for larger prospective studies in African diabetics to evaluate this as well as its cost effectiveness in resource poor-settings. 

## Figures and Tables

**Table 1:** Sociodemographic and clinical characteristics.

	Diabetics (*n* = 30)	Controls (*n* = 67)	*P*
Age (years)	37.8 ± 8.6	37.3 ± 9.9	0.78
Female gender	16 (40%)	24 (60%)	0.11
Systolic blood pressure (mmHg)	108 ± 6.5	110 ± 5.9	0.12
Diastolic blood pressure (mmHg)	77 ± 7.5	72.3 ± 7.2	0.01
Plasma glucose (mmol/L)	12.2 (8.5–14.8)	4.9 (4.3–5.6)	<0.001*
Serum creatinine (*μ*mol/L)	83.6 (81.8–133)	75.9 (75.9–101.3)	0.05*
Urinary creatinine (mmol/L)	12.8 ± 2.4	13.6 ± 3.7	0.19
eGFR (mL/min)	85.1 (45.4–111.8)	94.3 (73.8–127.7)	0.04*
Albumin/creatinine ratio (mg/mmol)	6.18 (4.68–9.42)	2.09 (1.51–2.81)	<0.001*

*Wilcoxon rank sum test for comparison of median (Interquartile range, IQR).

**Table 2:** Univariate and multivariate regression models for factors associated with urinary albumin/creatinine ratio.

	Univariate *β* (95% CI) *P* value	Multivariate *β* (95% CI) *P* value
NAG/creatinine ratio	**0.29 (0.24–0.34) <0.001**	**0.24 (0.18–0.30) <0.001**
Positive diabetic status	**4.61 (3.12–6.10) <0.001**	**3.05 (1.12–4.98) 0.002**
Age (years)	**0.02 (−0.06–0.11) 0.59**	**0.005 (**−**0.05–0.06) 0.87**
Male gender	−1.46 (−3.08–0.17) 0.08	−0.52 (**−**1.58–0.54) 0.32
Serum creatinine	0.01 (−0.02–0.04) 0.39	0.02 (−0.002–0.03) 0.08
Systolic blood pressure	−**0.07 (**−**0.21–0.06) 0.27**	**0.02 (−0.07–0.10) 0.73**
Diastolic blood pressure	**0.16 (0.06–0.26) 0.003**	**0.08 (0.01–0.15) 0.03**
